# Zero‐fluoroscopy transseptal puncture guided by right atrial electroanatomical mapping combined with intracardiac echocardiography: A single‐center experience

**DOI:** 10.1002/clc.23401

**Published:** 2020-06-07

**Authors:** Guangping Zhang, Liting Cheng, Zhuo Liang, Junmeng Zhang, Ruiqing Dong, Fei Hang, Xinlu Wang, Ziyu Wang, Lin Zhao, Zefeng Wang, Yongquan Wu

**Affiliations:** ^1^ Department of Cardiology Beijing Anzhen Hospital, Capital Medical University Beijing China; ^2^ Affiliated Hangzhou First People's Hospital Zhejiang University School of Medicine Hangzhou China; ^3^ Department of Lung and Blood Vessel Disease Beijing Institute of Heart Beijing China

**Keywords:** catheter ablation, intracardiac echocardiography, paroxysmal atrial fibrillation, right atria electroanatomical mapping, zero fluoroscopy

## Abstract

**Background:**

Right atrial electroanatomical mapping may be combined with SoundStar 3D diagnostic ultrasound catheter (EAM‐ICE) as a zero‐fluoroscopy procedure for radiofrequency catheter ablation (RFCA). We aimed to evaluate the efficiency and safety of zero‐fluoroscopy transseptal puncture guided by EAM‐ICE and fluoroscopy combined with intracardiac echocardiography (F‐ICE) in patients with paroxysmal atrial fibrillation (PAF).

**Hypothesis:**

Zero‐fluoroscopy transseptal puncture is an effective and safe procedure.

**Methods:**

This study had a prospective design. A total of 57 patients with PAF were enrolled and assigned to two groups. Twenty‐seven patients were enrolled in the EAM‐ICE group, and 30 patients were enrolled in the F‐ICE group.

**Results:**

There were no statistically significant differences in baseline patient characteristics between groups. Transseptal puncture was successful in all patients (57/57, 100%). Total procedure time and duration of transseptal puncture were lower in the F‐ICE group (199.4 ± 26.0 minutes vs 150.7 ± 22.1 minutes, *P* = 0.000; 118.4 ± 19.7 vs 70.5 ± 13.5 minutes, *P* = 0.000). There was no use of fluoroscopy in the EAM‐ICE group (0 mGy vs 70.5 ± 13.5 mGy); the duration of fluoroscopy in the EAM‐ICE group was negligible (0 minutes vs 5.4 ± 1.9 minutes). No procedural complication occurred in either group.

**Conclusions:**

EAM‐ICE guided zero‐fluoroscopy transseptal puncture is an effective and safe procedure.

## 1 INTRODUCTION

Fluoroscopy is used to perform electrophysiological studies and catheter ablations. However, radiation exposure can threaten the health of both doctors and patients.^1^, ^2^ Furthermore, radiation protection suits are heavy and may cause doctors musculoskeletal injury. The “as low as reasonably achievable” principle advocates limited radiation usage and proposes several methods to reduce radiation exposure,^3^ such as zero‐fluoroscopy procedures. Zero‐fluoroscopy procedures have recently become feasible because of the development of contact force‐sensing catheters, electroanatomical mapping systems (EAMs), and intracardiac echocardiography (ICE) probes.^4^ ICE‐guided radiofrequency catheter ablation (RFCA) has been studied in this context.^5^, ^6^, ^7^ However, few studies have investigated the application of ICE to procedures involving the atrial septum.

The foramen ovale is a unique anatomical structure located in thick muscle. During EAM, electrography yields low‐voltage readings for the foramen ovale. Whether this information can increase the success of transseptal puncture remains to be determined. No relevant studies have been conducted in Chinese populations. In this study, we developed a method for the combination of EAM with SoundStar 3D diagnostic ultrasound catheter (EAM‐ICE) to perform circumferential pulmonary vein isolation (CPVI). The efficacy and safety of EAM‐ICE‐guided transseptal puncture and catheter ablation are discussed below.

## 2 MATERIALS AND METHODS

### 2.1 Study design and population

A prospective, controlled trial was conducted at Beijing Anzhen Hospital. During the period from June 2019 to October 2019, 57 consecutive patients with paroxysmal atrial fibrillation were enrolled in the study. Patients were divided into two groups.

This study was approved by the Beijing Anzhen Hospital Medical Ethics Committee. All participants evaluated in this trial provided written informed consent.

The inclusion criteria were as follows: (a) adults aged 18 to 80 years; (b) previous diagnosis with paroxysmal atrial fibrillation (PAF), defined as AF terminating spontaneously or AF onset <7 days before RFCA, with verification of AF using 12‐lead electrography or Holter monitoring; (c) symptomatic PAF patients intolerant or refractory to antiarrhythmic medication or amenable to RFCA treatment.

The exclusion criteria were as follows: (a) left atrial appendage thrombosis; (b) abnormal cardiac structure disease (ie, severe mitral, tricuspid, or aortic malformations; atrial and ventricular septal defects; and tetralogy of Fallot); (c) heart failure; (d) history of RFCA, cryoballoon ablation, and/or cardiac surgery; (e) mental disorder; (f) eGFR <30 mL/min; (g) septic shock; (h) advanced malignant tumor; (i) pregnancy; (j) cardiac tamponade or major hydropericardium; and (k) left atrial anteroposterior diameter > 50 mm.

### 2.2 Procedure characteristics

Procedures were performed under local anesthesia. Electrode placement, transseptal puncture, and CPVI were performed according to standard protocols in each patient.

#### 2.2.1 EAM‐ICE guided transseptal puncture

Subjects in the EAM‐ICE group underwent transseptal puncture directed with use of the 3D EAM system (Carto; Biosense Webster, Diamond Bar, CA) and ICE (Soundstar; Biosense Webster) without the use of fluoroscopy.

1. Preparation

Bilateral femoral venous puncture was performed. An 8‐F introducer sheath (Input; Medtronic, Minneapolis, MN) was inserted in the right femoral vein; a 6‐F introducer (Input; Medtronic, Minneapolis, MN); and an 11‐F introducer sheath (Cordis, AVANTI; Ireland) were inserted in the left femoral vein. An ablation catheter (Thermocool Smarttouch; Biosense Webster) was inserted into the right atrium (RA) via the 8‐F introducer sheath.

2. Mapping the right atrium and foramen ovale

**FIGURE 1:**
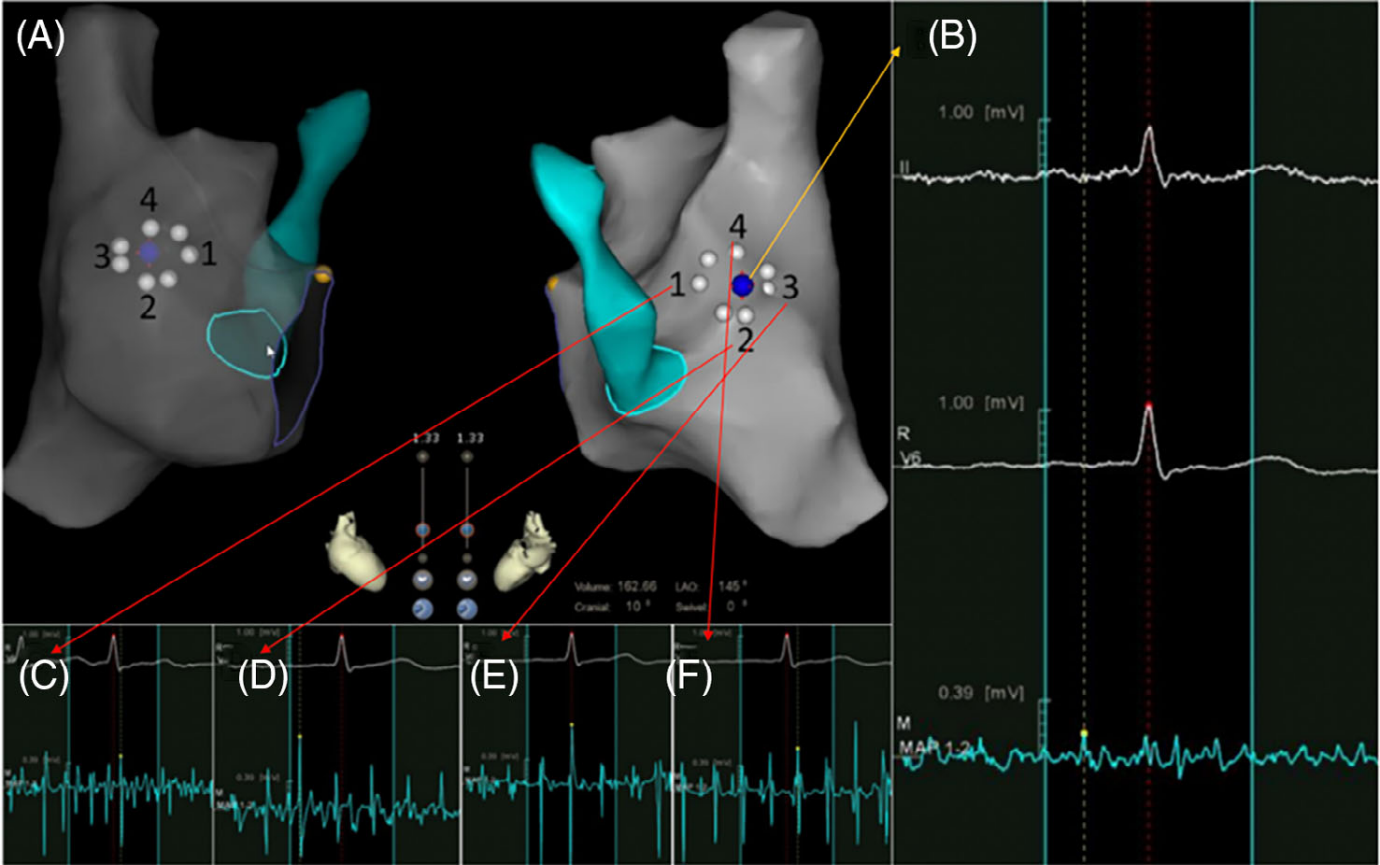
Foramen ovale mapping. A, An ablation catheter and EAM system were used to map the RA and the CS. The low‐voltage zone (foramen ovale) was marked on the anatomy (blue point). The circle formed by white points represents the foramen ovale as revealed by EAM. B, Electrography of the atrial septum was <0.3 mV (blue point). C to F. Electrography of the atrial muscle surrounding the septum. (C, electrogram of zone 1 in A; D, electrogram of zone 2 in A; E, electrogram of zone 3 in A; and F, electrogram of zone 4 in A). The magnitude of the electrogram was >0.5 mV. (yellow point, bundle of His; white point, foramen ovale area as revealed by voltage mapping; blue point, atrial septum; white point, area of low voltage identified by voltage mapping; yellow point, bundle of His; green area/circle, coronary sinus; EAM, electroanatomical mapping; RA, right atrium; CS, coronary sinus)

**FIGURE 2:**
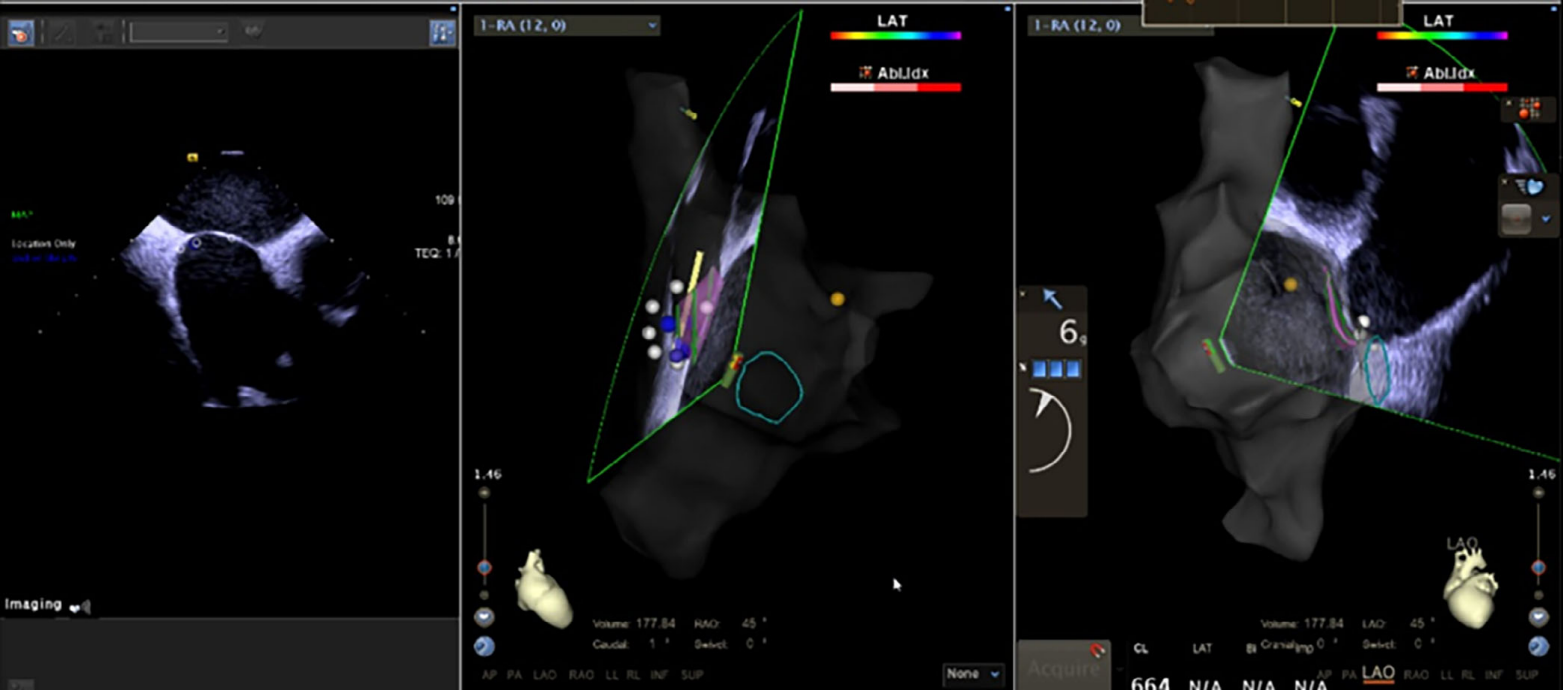
Intracardiac echocardiography (ICE) was performed to confirm the position of the foramen ovale. An ablation catheter and electroanatomical mapping systems (EAM) were used to map the anatomy of the foramen ovale. The position of the foramen ovale was confirmed from two readings. (pink area, foramen ovale as mapped by ICE; yellow point, bundle of His; white point, area of low voltage identified by voltage mapping; green area/circle, coronary sinus)

Inferior vena cava (IVC), superior vena cava (SVC), His bundle, tricuspid valve annulus, coronary sinus (CS), and the low‐voltage area in the atrial septum (foramen ovale) were carefully marked (Figure 1). Using the map, a 10‐polar diagnostic catheter was placed inside the CS. The ICE probe was then placed inside the RA using an 11‐F introducer. The position of the atrial septum was confirmed by ICE (Figure 2).

3. Left atrial mapping

Under the direction of ICE and EAM, a three‐dimensional (3D) left atrium (LA) map was created to visualize the anatomy of the LA and pulmonary veins. Not only the pulmonary veins and their ostia but also the left atrial appendage (LAA) was carefully mapped. We also checked the LAA for thrombosis.

4. Advancing the transseptal sheath into the SVC

Before inserting the J‐type guide‐wire into the SVC, we measured the distance from the site of puncture to the junction where the sternum met the second rib. We also marked the same distance on the non‐steerable SL1 sheath (Agilis TM, Fast Cath, Abbott, Abbott Park, IL). A clip‐pin cable was used to connect the J‐type guide‐wire to the Carto system for navigation. One side of each connector was clipped onto the guide‐wire; the other side was connected to the pinbox. The tip of the guide‐wire could thus be visualized on the Carto system as a bipolar electrode. Under the direction of ICE, the guide‐wire was inserted into the SVC. A non‐steerable SL1 sheath (Agilis TM, Fast Cath, Abbott). When slightly withdrawing the guide‐wire, bipolar electrode would disappear on Carto system, this position was marked as the tip of the sheath. After completely withdrawing the guide‐wire, a bolus of physiological solution was injected to confirm the position of the SVC. After rotation of the ICE probe, it was placed in a section facing the atrial septum.

5. Transseptal puncture

A clip‐pin cable was used to connect the transseptal needle (BRK‐1, St. Jude Medical, St. Paul, MN) to the Carto system. One side of each connector was clipped onto the transseptal needle; the other side was connected to the pinbox. Then the transseptal needle was placed inside the long sheath. The needle indicator was one thumb's width (approximately 1.5 cm) away from the inner sheath. The sheath was withdrawn slightly until the tip of the needle could be visualized as a bipolar electrode on the Carto system.

**FIGURE 3:**
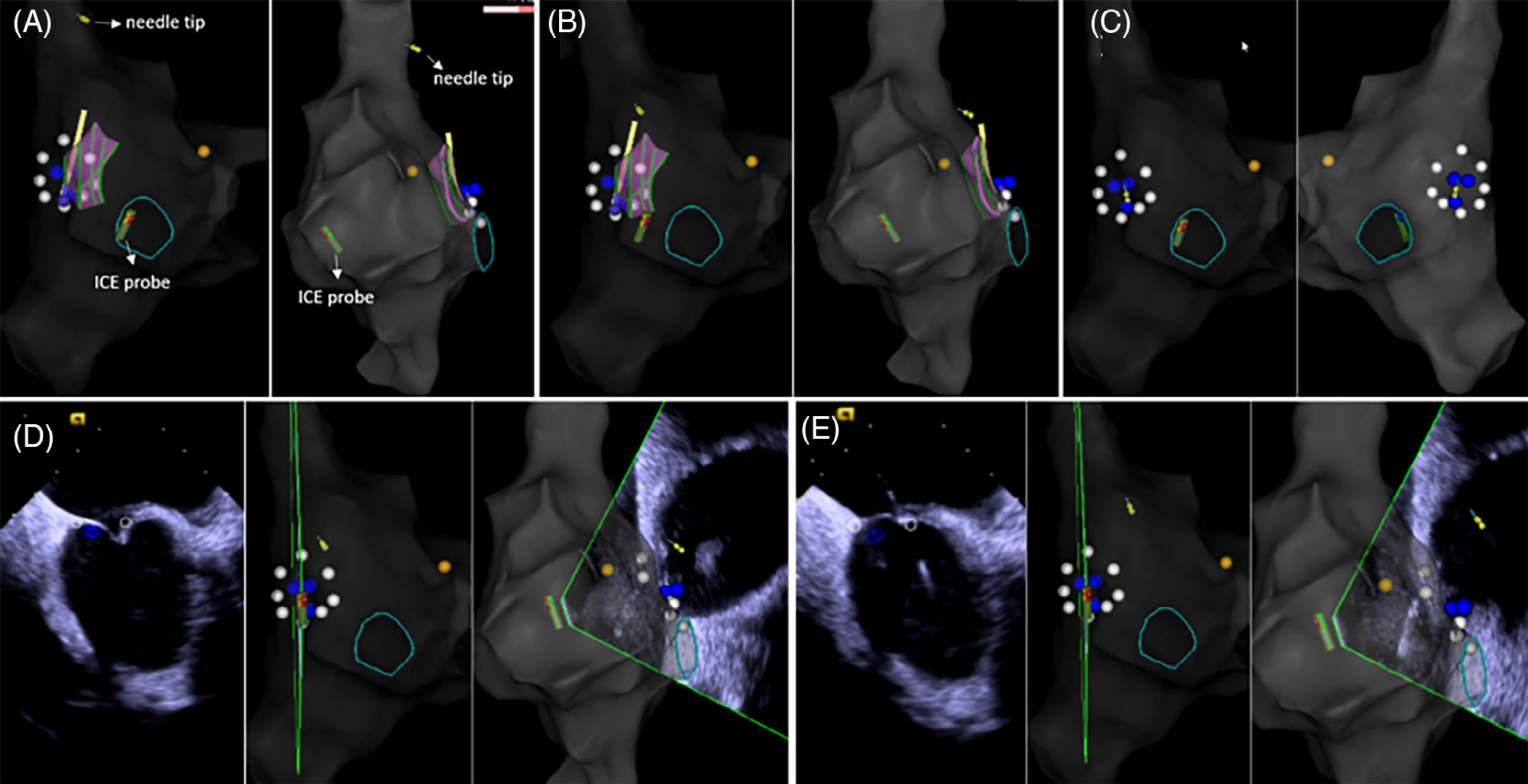
Transseptal puncture with electroanatomical mapping combined with diagnostic ultrasound catheter (EAM‐ICE). A, The long sheath was placed into the SVC through the guide‐wire, which was then replaced with the trans‐septal needle. The tip of the needle was inserted into the SVC (yellow circle). B and C, After withdrawal of the sheath‐dilator‐needle assembly, the tip the needle was oriented toward the atrial septum. The needle can be seen facing toward the septum in C. D, Under ICE direction, the trans‐septal needle was used to puncture the atrial septum. When the trans‐septal needle was pushed toward the atrial septum, a tenting phenomenon could be seen on ICE. E, After a rapid movement of the transseptal needle through the dilator succeeded in puncturing the atrial septum, a small bolus of physiological solution was used to confirm that the needle was inside the left atrium. The shadow created by the bolus can be seen on ICE. (yellow circle, tip of the trans‐septal needle; yellow point, bundle of His; white point, foramen ovale; blue point, low‐voltage area)

Under the direction of the 3D EAM system, the sheath‐dilator‐needle assembly was pulled out by rotating it in a clockwise direction until it faced the RA septum. Tenting of the interatrial septum by the sheath‐dilator‐needle was noted on ICE. The transseptal needle was then pulled rapidly through the dilator to puncture the atrial septum. The routine of sheath‐dilator‐needle assembly can be visualized on 3D‐EAM (Figure 3). Three methods were used for confirmation: first, syringe withdrawal. If the puncture was successful, blood gently flowed into the syringe. Second, a bolus of physiological solution was used to confirm the puncture. A shadow of the solution bolus was visualized with ICE. Third, a pressure transducer was used to evaluate puncture success. After a successful puncture had been confirmed, the sheath‐dilator assembly was advanced over the needle. After needle withdrawal, a long wire was inserted across the LA into the left superior pulmonary vein (LSPV). The sheath‐dilator was advanced into the LA. The dilator and long wire were withdrawn. Then, a bolus of physiological solution was injected through the sheath. A shadow of the solution bolus visualized on ICE provided confirmation that the LA had been penetrated. The ablation catheter was advanced inside the LA (Figure 3).

#### 2.2.2 Fluoroscopy‐guided transseptal puncture

For patients in the F‐ICE group, the procedure was performed under fluoroscopic and ICE guidance.

1. Preparation

To access the heart, bilateral femoral venous puncture was performed. A guide‐wire for the long sheath was inserted into the right femoral vein; a 6‐F introducer and an 11‐F introducer sheath were inserted into the left femoral vein. ICE was used to visualize the anatomy of the foramen ovale on the side facing the left pulmonary vein. Then, a 10‐polar diagnostic catheter was inserted into the CS under fluoroscopic guidance.

2. Left atrial mapping

Under the direction of ICE and EAM, a 3D LA map was created to visualize the anatomy of the LA and pulmonary veins.

3. Transseptal puncture

A transseptal puncture was then performed. A long sheath was delivered into the SVC through the guidewire under fluoroscopic guidance. The guidewire was then removed and replaced with a transseptal needle. The distance between the tip of the transseptal needle and the tip of the long sheath was decreased by 0.5 to 1 cm. The transseptal needle and long sheath were withdrawn simultaneously, and the sheath tip was observed to jump, twice. After the second jump, the tip was pointed toward the foramen ovale. We used RAO 45° to confirm that the tip of the sheath was vertical to the atrial septum. Sheath tip location and tenting were confirmed with ICE. The transseptal needle was then used to puncture the atrial septum. Four methods were used for confirmation: first, needle withdrawal. If the puncture was successful, blood gently flowed into the syringe. Second, a small bolus of physiological solution was injected to confirm puncture success. The shadow created by the bolus was observed on ICE. Third, a pressure transducer was used evaluate whether the puncture had been successful. Fourth, contrast medium was injected through the needle, and a thick, dark line could be seen on fluoroscopy. After confirmation, the dilator inside the long sheath was pushed through the transseptal needle into the LA. Next, the needle was withdrawn, and the long wire was inserted into the LSPV. Transseptal punctures were performed once, and a non‐steerable long sheath was inserted into the LA. A 3D LA map was created with ICE to visualize the anatomy of the LA and pulmonary veins.

#### 2.2.3 Catheter ablation

Point‐by‐point CPVI was performed using irrigated ablation catheters (Thermocool Smarttouch; Biosense Webster) in power control mode at 35 W (irrigation flow 17 mL/min). Radiofrequency applications were performed with Visitag (Carto, Biosense Webster) guidance with catheter stability (2.5 mm for 3 seconds) and contact force (CF; >3 *g* for 25% of time) settings. The ablation index (AI) targeted at the anterior LA wall was 500, and the AI targeted at the posterior wall was 400. During ablation, an ICE probe was placed in the right ventricle to monitor the pericardium. Heparin was given during the procedure to maintain an activated clotting time between 300 and 350.

### 2.3 Baseline evaluation

Patients' baseline characteristics (ie, age, sex, body mass index [BMI], and past history) were assessed. Echocardiographic findings (ie, left ventricle ejection fraction, LA anteroposterior diameter) were also recorded.

### 2.4 Randomization, treatment allocation, and outcomes

Subjects fitting the inclusion and exclusion criteria were assigned to the EAM‐ICE or F‐ICE group. The primary outcome of this trial was the rate of successful transseptal puncture. The secondary outcomes of this trial were the rates of major complications (ie, cardiac perforation, hydropericardium, malignant arrhythmia, sudden cardiac death, atrial esophageal fistula, and acute myocardial infarction).

### 2.5 Statistical analysis

Data analysis was performed with SPSS statistical software (IBM, Version 23). Normally distributed continuous variables are expressed as means ± SD; non‐uniformly distributed data are expressed as medians (Q1 and Q3). Between‐group comparisons of means were analyzed by independent‐samples *t* test for normally distributed data and by Mann‐Whitney *U* test for non‐uniformly distributed data. A *P*‐value <0.05 was considered statistically significant.

## 3 RESULTS

### 3.1 Baseline characteristics

This study included 57 patients, 42 (73.7%) of whom were male. A total of 27 (47.4%) patients were enrolled in the EAM‐ICE group. Patients' detailed information is shown in Table 1. There were no statistically significant differences in baseline patient characteristics between groups.

**TABLE 1:** Baseline patient characteristics

	EAM‐ICE (n = 27)	F‐ICE (n = 30)	*P* value
Age, y	58.1 ± 11.3	62 ± 9.6	0.171
Male, n (%)	21(77.8%)	21 (70%)	0.506
BMI, kg/m^2^	25.6 ± 2.9	26.1 ± 9.3	0.666
AF duration, months	20 (10, 28)	24 (12, 36)	0.237
Hypertension, n (%)	14 (51.9%)	18 (60%)	0.536
Diabetes, n (%)	5 (18.5%)	6 (20%)	0.887
CHD, n (%)	2 (7.4%)	2 (6.7%)	0.913
CHA_2_DS_2_‐VAS_C_ ≥ 2, n(%)	12 (40%)	12 (44.4%)	0.237
LAAP‐diameter, mm	39.7 ± 5.1	37.7 ± 4.8	0.120
EF, %	63.1 ± 4.2	63.8 ± 4.5	0.571

Abbreviations: AF, atrial fibrillation; BMI, body mass index; CHD, coronary heart disease; EF, ejection fraction; LAAP diameter, left atrial anteroposterior diameter.

### 3.2 Procedural data

We divided the procedure of transseptal puncture into several steps to evaluate the differences between groups.

### 3.3 Outcomes

Trans‐septal puncture was successful in all cases included in the study (57/57, 100%). We also evaluated total procedure time and trans‐septal duration in the EAM‐ICE and F‐ICE groups. Total procedure duration was defined as the duration of the patient's stay in the operating room. In the EAM‐ICE group, the duration of trans‐septal puncture was considered to be the time required for mapping the RA and foramen ovale with EAM, mapping the LA and foramen ovale with ICE, advancing the long wire and sheath into the SVC, and transseptal puncture. In the F‐ICE group, the duration of transseptal puncture included LA mapping, advancing the long wire and sheath into the SVC, and transseptal puncture. Although total procedure time and transseptal duration were lower in the F‐ICE group (199.4 ± 26.0 minutes vs 150.7 ± 22.1 minutes, *P* = 0.000; 118.4 ± 19.7 vs 70.5 ± 13.5 minutes, *P* = 0.000), there was no use of fluoroscopy in the EAM‐ICE group (0 mGy vs 70.5 ± 13.5 mGy), and fluoroscopy time was negligible in the EAM‐ICE group (0 minutes vs 5.4 ± 1.9 minutes). No procedural complication (ie, cardiac perforation, hydropericardium, malignant arrhythmia, sudden cardiac death, atrial esophageal fistula, and acute myocardial infarction) occurred in either group (Table 2).

**TABLE 2:** Outcomes

	EAM‐ICE (n = 27)	F‐ICE (n = 30)	*P* value
Total procedure duration^a^, min	199.4 ± 26.0	150.7 ± 22.1	0.000
Transseptal duration^b^, min	118.4 ± 19.7	70.5 ± 13.5	0.000
Ablation duration^c^, min	48.4 ± 6.1	48.7 ± 5.5	0.814
Radiation dosage, mGy	0	70.5 ± 13.5	0.000
Fluoroscopy time, min	0	5.4 ± 1.9	0.000

Abbreviations: EAM‐ICE, electroanatomical mapping combined with diagnostic ultrasound catheter; F‐ICE, fluoroscopy combined with intracardiac echocardiography.

a Total procedure duration was defined as the duration of the patient's stay in the operating room.

b In the EAM‐ICE group, trans‐septal duration included mapping the right atrium (RA) and foramen ovale with EAM, mapping the left atrium (LA) and foramen ovale with ICE, advancing the long wire and sheath into the SVC, and transseptal puncture. In the F‐ICE group, transseptal duration included LA mapping, advancing the long wire and sheath into the SVC, and transseptal puncture.

c The duration of ablation duration was defined as the duration of circumferential pulmonary vein isolation (CPVI).

### 3.4 EAM vs ICE for mapping the foramen ovale mapping

We compared the anatomical structures revealed by foramen ovale mapping in the EAM‐ICE groups. The visualizations of anatomical structure obtained with EAM and ICE were in agreement in all groups.

## 4 DISCUSSION

Based on the results presented above, we conclude that EAM‐ICE is an effective and safe procedure. Thus, a zero‐fluoroscopy procedure is feasible. Fluoroscopic guidance is used to perform electrophysiological studies and catheter ablations. Despite the fact that the procedure can improve the quality of life for patients, radiation exposure can lead to malignancies, genetic defects, skin injury, cataract, and other complications, with the potential for negative effects on the health of doctors and patients.^1^, ^2^ With the development of contact force‐sensing catheters, EAM, transesophageal echocardiography (TEE), and ICE probes, zero‐fluoroscopy procedures have received increased attention from electrophysiologists.^4^, ^7^, ^8^

Transseptal puncture is a key step of this procedure. With use of the transseptal puncture method, zero‐fluoroscopy methods can be performed in two ways, with TEE or ICE. However, with the use of TEE, patients have to be sedated, and the procedure is expensive because of the additional cost of anesthesia. Additionally, TEE can cause damage to the esophageal mucous membrane. The ablation energy delivered to the posterior wall of the LA may also aggravate esophageal lesions, increasing the incidence of atrial esophageal fistula and risk for life‐threating complications. Studies have also shown that RFCA can be performed under the direction of ICE.

In recent studies, the tenting phenomenon that occurs during ICE was used to direct transseptal puncture.^9^, ^10^ Baykaner et al.^11^ performed zero‐fluoroscopy transseptal puncture under the direction of ICE by placing the ablation catheter onto the atrial septum, which created mild tenting and allowed the position of the sheath to be confirmed. The authors of the study then replaced the ablation catheter with a transseptal needle and performed transseptal puncture. However, ICE can only provide information in one sector and does not include detailed information about the atrial septum. The position of the ICE probe often requires adjustment during transseptal puncture to ensure that the angle is correct. The ICE probe cannot be used effectively to follow the movement of the sheath‐dilator‐needle assembly, which prevents visualization of the dynamic movement of the assembly or a pin attached to the tip. The information ICE provides is insufficient for operators, and use of ICE decreases the safety of associated procedures. The use of fluoroscopy in combination with ICE improves short‐term outcomes. F‐ICE guidance provides two‐dimensional guidance. The specific position of a given anatomical structure has to be deduced from bone structure or from the contrast between heart and lung. Nevertheless, the procedure is highly demanding for operators. Above all, a more effective and safe method is needed.

Three‐dimensional EAM has been used in association with catheter ablation for atrioventricular nodal reentrant tachycardia, cavotricuspid isthmus‐dependent atrial flutter, and premature ventricular contraction, especially in pediatric and pregnant patients.^12^, ^13^, ^14^, ^15^, ^16^, ^17^ Three‐dimensional EAM holds promise as a tool for use in combination with transseptal puncture. The RA is located anterior to the rest of the heart, which places septum structures at an angle of approximately 65°, with respect to the right posterior heart. This angle may change due to enlargement of the RA or LA.^18^ The atrial septum, which separates the atrial chambers, contains a foramen ovale with an infolded rim. The foramen ovale is commonly used for transseptal puncture. It is formed by a layer of thick muscle that appears as an area of low voltage on structural maps. Whether this characteristic can direct transseptal puncture remains to be elucidated. Clark et al. reported a cohort study in which EAM was used in combination with ICE to perform transseptal puncture. By pulling down the ablation catheter, the authors located the position of the foramen ovale.^17^ However, the advantages of EAM were not fully utilized in their study. Haegeli et al. reported one case of transsptal puncture directed by 3D EAM without the guidance of ICE. The authors dragged the ablation catheter, which allowed the tip of the catheter to jump to the region of the foramen ovale.^19^ However, this manuscript did not provide detailed information.

Base on this anatomical characteristic, we decided to perform zero‐fluoroscopy RFCA by combining EAM and ICE. After the EAM system was used to direct RA mapping, a low‐voltage area was mapped in the area of the atrial septum. This low‐voltage area is an optimal location for transseptal puncture. In all cases presented above, transseptal puncture was conducted successfully without the use of fluoroscopy. The fact that no complications occurred during EAM‐ICE reinforces the safety of the procedure, indicating that EAM‐ICE in non‐inferior to F‐ICE. After transseptal puncture, RFCA was performed with ICE guidance.

Procedure time was longer in the EAM‐ICE group (199.4 ± 26.0 minutes vs 150.7 ± 22.1 minutes, *P* = 0.000). There are several reasons for this prolonged duration. First, we used high‐density voltage mapping to locate the foramen ovale, which required approximately 25 minutes. The foramen ovale was recognized as a low‐voltage zone in the RA. Because we wanted to test the theory that fluoro‐less trans‐septal puncture may be performed without the guidance of ICE, the foramen ovale was initially mapped only with voltage mapping. We enrolled the first few cases treated in this manner in the study. Prolonged trans‐septal durations may therefore indicate unskilled or extremely cautious attempts to map the foramen ovale. For these reasons, the duration of trans‐septal puncture was greater than the duration of RA mapping alone. We also established a special technique for transseptal puncture. By using a clip‐pin cable, we were able to connect the guide‐wire and transseptal needle to the Carto system, which allowed us to visualize the tip of this assembly as a bipolar electrode. This procedure, along with the necessary adjustments, required 15 minutes. During the procedure, an ICE probe was used to verify that the technique described was feasible for mapping the anatomy of the foramen ovale, coronary sinus, and LA and to ensure that the procedure was safe. In contrast, in the F‐ICE group, “trans‐septal duration” was defined as the time required for trans‐septal puncture guided by fluoroscopy, added to the time required for LA mapping guided by ICE. All members of our team are proficient at this technique. Procedure time is likely to decrease as operators become more skilled in performing the required techniques.

To evaluate the differences between procedures, we divided transseptal puncture into several steps. First, we evaluated the accuracy with which the anatomical structures of the RA, LA, SVC, and foramen ovale were visualized. With EAM‐ICE, anatomical structures can be displayed from various angles; however, with F‐ICE, anatomical structures can only be displayed with fluoroscopy, which requires that a specific position be deduced from bone landmarks. The foramen ovale is particularly difficult to recognize with F‐ICE because it can only be evaluated in one ICE sector at a time. We also evaluated whether the tip of the sheath‐dilator‐needle assembly could carry a pin. EAM‐ICE traced the needle tip in three dimensions, while F‐ICE traced the needle tip in two dimensions. EAM‐ICE provided more information than ICE by providing details pertaining to anatomical structures, rendering the procedure effective and safe. Third, when withdrawing the transseptal needle and long sheath together, movement can be evaluated dynamically using either technique. In both groups, two episodes of jumping were discernible.

EAM‐ICE upgrades the function of ICE by providing 3D visualization of anatomical structures, thus making the accompanying procedure more effective and safer.

## 5 LIMITATIONS

We only analyzed acute outcomes. AF recurrence and long‐term success rates were not analyzed in this trial, which limits the strength of our findings. Patients were not grouped in a random fashion, which limits the strength of the conclusions made from comparisons between EAM‐ICE and F‐ICE.

## 6 CONCLUSION

It is effective and safe to perform the EAM‐ICE procedure. Thus, the use of a zero‐fluoroscopy approach for PAF is feasible.

## CONFLICT OF INTEREST

The authors declare no potential conflict of interests.
